# Growth Velocity of Infants from Birth to 5 Years Born in Maku, Iran

**DOI:** 10.5539/gjhs.v8n2p56

**Published:** 2015-06-05

**Authors:** Seyed Mohammad Taghi Ayatollahi, Elham Haem, Zahra Sharafi

**Affiliations:** 1 Department of Biostatistics, Shiraz University of Medical Sciences School of Medicine, Shiraz, Iran

**Keywords:** growth, velocity, weight, height, head circumference

## Abstract

**Background:**

Growth velocity standards are essential for proper evaluation of child growth. The goal of this study was to construct weight, height and head circumference growth velocity charts for infants.

**Methods:**

This study includes 256 infants (124 boys and 132 girls) born in Maku, Northwest of Iran, and monitored from birth until they were 5 years. The weights and heights of the subjects were recorded at birth, one, two, four, six months and 1, 1.5, 2, 3, 4 and 5 years of age, while the head circumferences were measured until they were 1.5 years old. In this study, the LMS method using LMS chart maker software, was utilized to obtain growth velocity centiles.

**Results:**

Growth velocity charts for weight, height and head circumference (5th, 50th, 95th percentiles) were obtained. The velocity growth charts decreased rapidly from birth to 2 years and then remained relatively constant up to 5 years for both sexes. The growth velocity of boys was higher than girls through the first year of age but became equal at 12 months of age and no significant difference was seen up to 5 years.

**Conclusion:**

Growth velocity studies are really sparse in Iran. In this study, longitudinal data were used to obtain growth velocity centiles. Furthermore, the weight and height velocities of infants from Shiraz, southern Iran, and U.K were compared.

## 1. Introduction

Growth is a dynamic process whereby a living organism increases in size over a period of time. The accurate monitoring of physical growth in infants is of value for the assessment of health status, identifying deviations from normality and determining the effectiveness of interventions (de Onis et al., 2012).

Anthropometric measurements are significant indices to predict infant health and future outcome (S. M. Ayatollahi & Shahsavari, 2002). Weight, height and head circumference of new born infants are the most common parameters for measuring the physical growth of infants (Fok et al., 2003).

Growth velocity is a desirable approach that depicts the age-dependent changes in velocity that characterize human postnatal growth (De Onis & World Health Organization, 2009). Standards for velocity are required to determine whether the rate of infant growth over the past month, had been within normal limits for his or her age, sex, etc. It has advantages as an epidemiological tool when compared with distance charts. The latter is the result of a cumulative growth experience, whereas growth velocity represents what is happening at present (Hosseini, Maracy, Sarrafzade, & Kelishadi, 2014).

Several epidemiological studies have investigated growth velocity standards for children in America (de Onis et al., 2011; A. Roche, Guo, & Moore, 1989; A. F. Roche & Himes, 1980; Tanner & Davies, 1985), Europe (Gerver & de Bruin, 2003; Lampl & Jeanty, 2003; Michaelsen, Petersen, Greisen, & Thomsen, 1994; Tanner, Whitehouse, & Takaishi, 1966a, 1996b) and Asia (Chae et al., 2013; Costello, 1989; Kolsteren, Kusin, & Kardjati, 1997).

Several studies have assessed infant growth using distance charts and constructed reference growth curves during the past two decades in Iran (S. M. Ayatollahi & Shahsavari, 2002; S. M. T. Ayatollahi, 2001, 2012; S. Ayatollahi & Ahmadi, 2001; S. M. T. Ayatollahi & Shayan, 2008; Emdadi, Safarian, & Doosti, 2011; Heydari, 2009; Mirfazeli, Besharat, Rashedi, & Rabiee, 2009). Based on the longitudinal data however, the only reference growth velocity standard in Iran was provided by the first author (S. M. T. Ayatollahi, 2005) in Shiraz, one of the five main cities of Iran.

This paper presents growth velocity standards for height, weight and head circumference of a birth cohort, followed longitudinally from birth to 5 years of age in Maku, and compared the relevant anthropometric velocity measures with that of the study conducted in Shiraz (S. M. T. Ayatollahi, 2005).

## 2. Methods

Maku, one of the West Azerbaijan province cities with a rural population, is located in the Northwest of Iran, sharing borders with Turkey. It has a semi-arid cold climate and a human population of 202939, including 8.13% of the total population of the province (http://fa.wikipedia.org).

### 2.1 Subjects

A birth cohort of 256 apparently healthy neonates (124 boys and 132 girls) born in Maku in 2004 were recruited. The subjects were followed for five years until 2009 and visited at the health centers. A questionnaire on the demographic and health status of the neonates and their parents including anthropometric measurements was completed. The weighs and heights of the subjects were measured by trained auxologists at birth, one, two, four, six months and 1, 1.5, 2, 3, 4 and 5 years of age and head circumferences were recorded just up to 1.5 years of age. Heights were measured in supine position until one year of age and then in standing position in centimeter. Weights were measured to the nearest 10 g until the second year of age using a baby scale and onwards to 0.1 kg.

### 2.2 Statistical Analysis

The birth weight of 10 subjects (3.9%) were under 2500 g (range 1600-2400g). No subject was observed that failed to thrive. Only one subject left the study by age of 4 years and another 18 subjects (7%) by age of 5 years, giving a total attrition rate of 7.4%.

The growth velocity formula is given as
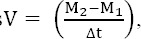
 where M1 and M2 are measurements at adjacent occasions, and Δ*t* is the time interval between them. Due to the fact that each measurement has its own measurement error, the variance V is given by var(V) = σ^2^ + 2ε^2^/Δt, where ε is the measurement error and σ is the population standard deviation of the true measurement velocity (Bairagi, 1986). This indicates that the variability of velocity (and hence the spacing of centile curves) depends on the time interval Δ*t* between measurements. The velocity standards must therefore be tied to a particular time interval, to ensure that the two components of error are weighted appropriately. For length and weight during infancy, intervals between 2 weeks and 3 months have been proposed (Bairagi, 1986; Emery, Waite, Carpenter, Limerick, & Blake, 1985; Guo et al., 1991; Piwoz, Peerson, & Brown, 1992).

Cole’s LMS method (Cole & Green, 1992) was performed to construct smoothed weight, height and head circumference velocity centiles. The LMS method summarizes the changing distribution using the median(M) curve, the coefficient of variation (CV) of the distribution (called the S curve) and the skewness of distribution, expressed as a Box-Cox power (the L curve) (Tanner, Whitehouse, & Takaishi, 1966a, 1996b).

Height is usually normally distributed, while most other anthropometric measurements tend to be skewed. The benefit of the LMS method is that the normalized growth centile standards are obtained using SD scores which deal with skewness that may be presented in the distribution of measurements, such as height or weight. The SD score (standard deviation score) or the Z score was calculated using the formula below:


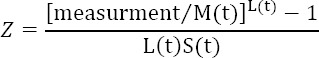


Where, measurement is weight, height or head circumference velocity for an infant of age t months (Bong, Shariff, Mohamed, & Merican, 2012). The LMS method assumes that skewed data can be normalized using a power transformation which stretches one tail of the distribution and shrinks the other tail simultaneously, thus removing the skewness. Using penalized likelihood the L, M and S curves can be fitted as cubic splines by non-linear regression, and the extent of smoothing required can be expressed in terms of smoothing parameters or equivalent degree of freedom. Finally, any required centile curve *C_100α_^(t)^* as specified by the corresponding Normal equivalent deviate Z_α_, for tail area α, could be derived from(Bong et al., 2012).


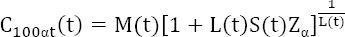


Statistical analysis was performed using statistical analysis software SPSS version 11.5 (SPSS Inc., Chicago, USA). LMS chart maker (professional) and Excel softwares were used for construction of weight, height and head circumference velocity centiles. Furthermore, the goodness of fit of data was evaluated by the Z-score method and by the Q-Q plot using the LMS chart maker software (Pan & Cole, 1997).

## 3. Results

**Figure 1:**
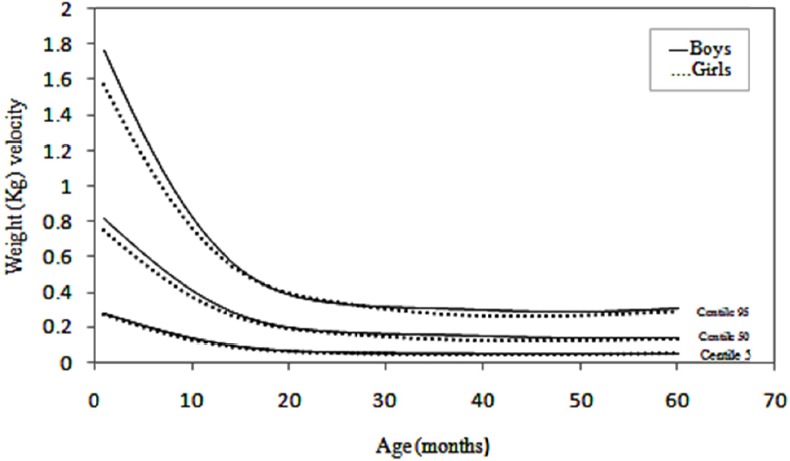
Weight (Kg) velocity of infants in Maku, Iran

**Figure 2:**
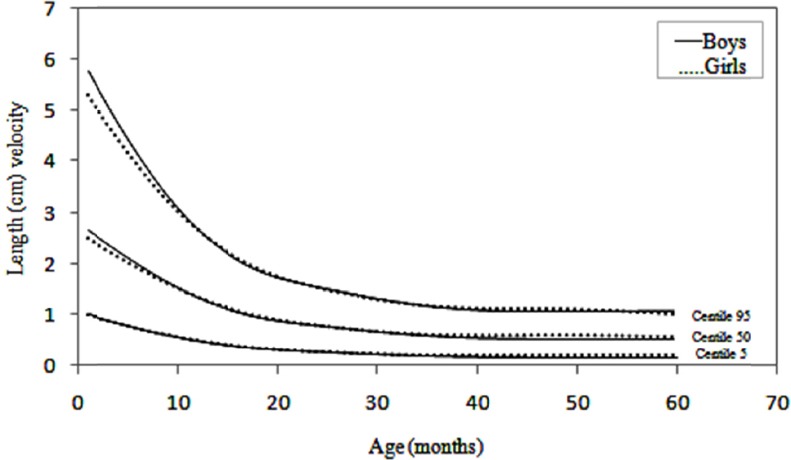
Length (cm) velocity of infants in Maku, Iran

**Figure 3:**
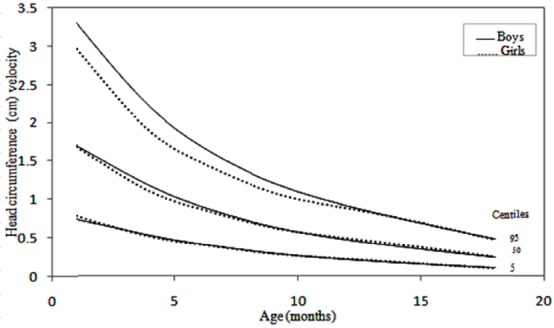
Head circumference (cm) velocity of infants in Maku, Iran

Changes in velocity are perhaps best appreciated in graphical form. Velocity charts are shown for both sexes between the ages of 1 and 60 months for weight and length (Figures 1 and 2) and Figure 3 presents head circumference velocity for both sexes between 1 and 18 months for median and extreme centiles.

## 4. Discussion

Since normal growth is a reflection of overall health and nutritional status, growth monitoring is essential for the proper assessment of the growth of children. Growth velocity charts are useful as screening tools to assess short-term changes in the growth rate of children. A normal growth velocity shows current good health in children, while a decrease in the growth velocity may indicate poor health and even onset of a chronic illness, such as inflammatory bowel disease.

This longitudinal study investigated a rural population of 256 neonates who were followed from birth up to 5 years. Growth velocities for weight, length and head circumference in boys and girls were investigated and the serial correlations obtained are as shown in Table 1. The small correlations between successive increments are beneficial for constructing velocity charts based on longitudinal data.

**Table 1:** Serial correlations of successive anthropometric measurement increments

Target month interval	weight		length		Head	

	Boys	Girls	Boys	Girls	Boys	Girls
**0-1 vs. 1-2**	-0.06	-0.01	0.02	0.18	-0.10	0.10
**1-2 vs. 2-4**	0.06	0.09	-0.25	-0.19	-0.07	-0.12
**2-4 vs. 4-6**	-.09	0.20	-0.18	-0.19	-0.12	<0.01
**4-6 vs. 6-9**	0.01	0.12	-0.12	-0.16	<- 0.01	0.07
**6-9 vs. 9-12**	-0.15	-0.22	0.02	-0.11	-.98	-0.06
**9-12 vs. 12-18**	-0.13	-0.04	0.02	-0.28	0.13	0.03
**12-18 vs. 18-24**	-0.26	-0.24	0.10	<0.01		
**18-24 vs. 24-36**	-0.10	-0.08	0.22	0.08		
**24-36 vs. 36-48**	0.06	<0.01	0.15	0.11		
**36-48 vs. 48-60**	0.15	0.25	0.30	0.12		

In the present study weight, length and head circumference velocity curves showed the highest values immediately after birth and decreased through the first 2 years and then remained relatively constant up to 5 years, as shown in Figures 1, 2 and 3. As illustrated, the male neonates grew slightly faster than the females, but the velocities equilibrated at 12 months of age. Then, up to 5 years there were no significant differences in growth rates.

The statistical method handled the primary difference between the present study and the previous one (S. Ayatollahi, 2005) for constructing velocity charts. In the present study, the parametric LMS method was used, this method has the advantage that after a suitable power transformation, the data are normally distributed and provide smoother centiles. Another advantage of the LMS method is that the three curves, L, M and S, completely summarize the measurement’s distribution over the range of covariates, and in addition they may be of interest in their own right. A key assumption of the LMS method is that after a suitable power transformation, the data becomes normally distributed. Anthropometric measurements, particularly weight and height, tend to follow this pattern (S. M. T. Ayatollahi, 2010). In the previous study (S. M. T. Ayatollahi, 2005), the nonparametric Healy-Rasbash-Yang (HRY) method (Healy, Rasbash, & Yang, 1988) was applied. Before applying this method on anthropometric measurements, for normalizing the variables, some transformation such as the log transformation should be applied.

**Figure 5:**
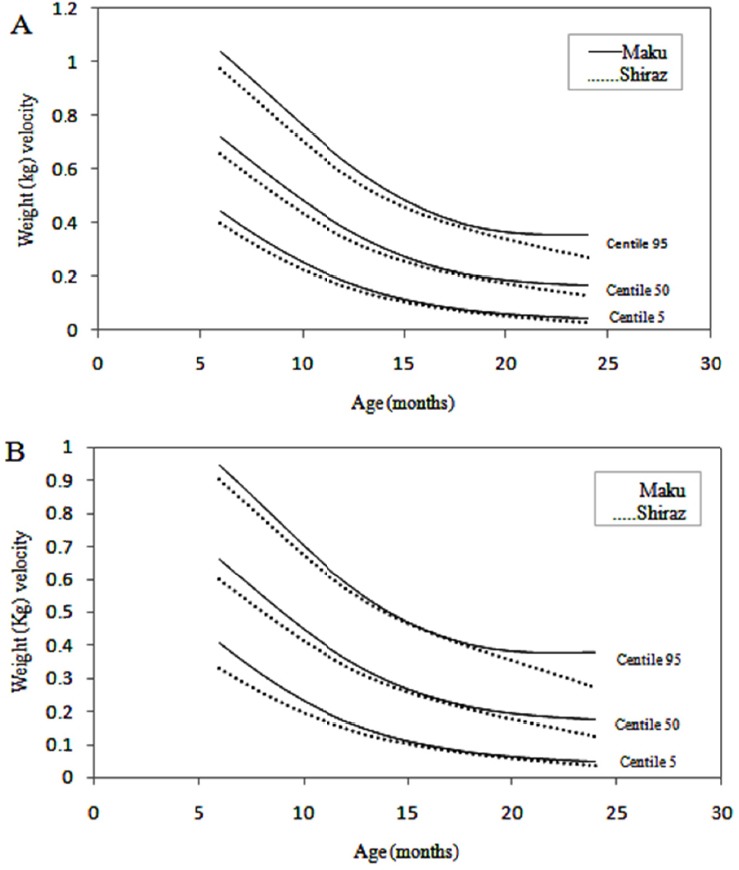
Weight (Kg) velocity of infants in Maku and Shiraz, Iran. A, Boys; B, Girls

**Figure 6:**
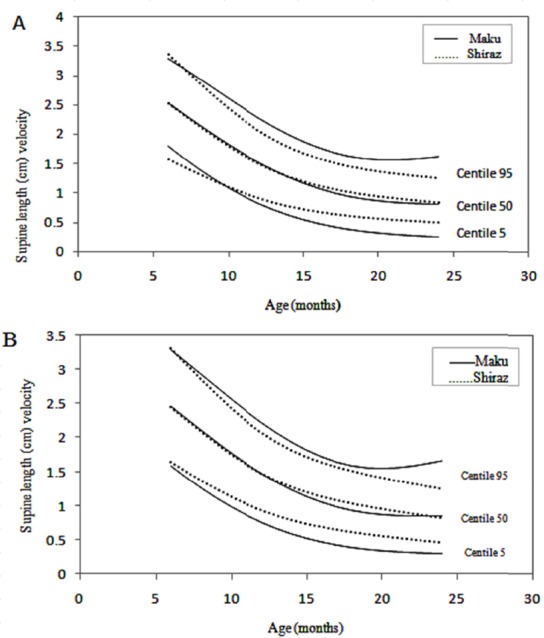
Supine length (cm) velocity of infants in Maku and Shiraz, Iran. A, Boys; B, Girls

In this study, the weight and height velocity charts of infants in Maku were compared with those in Shiraz (S. M. T. Ayatollahi, 2005) by the LMS method. Figures 5 and 6 compares the median and extreme centiles of weight and height of the velocities of Maku and Shiraz infants by sex and chronological age. The weight velocity standards of the Maku infants were slightly higher, when compared to Shiraz infants and was more pronounced in the first year of life. This result may be due to the difference between rural and urban lifestyles such as traditional and natural nutrition, the use of clean air and access to free health centers for the public.

In addition, the median of weight and height velocity of Iranian (Maku and Shiraz) infants were compared to corresponding U.K infants by sex and chronological age (Table 2). The result of this study confirms the previous finding (S. M. T. Ayatollahi, 2005) that the height velocity median of Tanner (J. Tanner et al., 1966a, 1996b) was lower than the Iranian one in the first year, while the weight velocity median was significantly higher than values obtained for the Iranian infants. The differences may be due to genetic factors, environmental factors, or both. Furthermore, the results of Table 2 are evident to sex differences in the velocity measurements in each of the three studies. It should be noted that the subjects were healthy infants and no widespread malnutrition was reported. As a result, the existing differences between rural and urban growth velocity standards, supports the fact that local growth standards should be applied for assessing growth status in Iran. The representativeness of the study’s sample in terms of demographic as well as socioeconomic homogeneity with the rural population of Iran, proposes that the growth velocity standards calculated for Maku infants are likely to be generally applicable to rural infants in Iran.

**Table 2:** Comparison of 50^th^ centile (median) of height and weight velocities of Iranian and U.K. infants by sex

	Height velocity (cm/yr)						Weight velocity (kg/yr)					

	Boys			Girls			Boys			Girls		
	Shiraz	Maku	U.K.	Shiraz	Maku	U.K.	Shiraz	Maku	U.K.	Shiraz	Maku	U.K.
First year	24.4	24.3	24.2	23.8	23.3	23	6.1	6.4	6.7	5.7	5.9	6.3
Second year	11.7	10.4	10.6	11.2	10.3	11.4	2.3	2.8	2.5	2.2	2.7	2.5

In conclusion, this research is the first report of growth velocity standards for weight, height and head circumference in Iranian rural infants, by analyzing longitudinal data with the parametric LMS method.
